# Complete genome sequence of a Chinese *Pseudomonas syringae* pv. *actinidiae* strain Yunnan3.2

**DOI:** 10.1128/mra.01070-23

**Published:** 2024-02-01

**Authors:** Guanglu Yao, Fenghuan Yang, Chao Yu, Qin Wang, Yijuan Yang, Huamin Chen

**Affiliations:** 1 Plant Protection and Quarantine Station of Zhaotong City, Zhaotong, China; 2 State Key Laboratory for Biology of Plant Diseases and Insect Pests, Institute of Plant Protection, Chinese Academy of Agricultural Sciences, Beijing, China; The University of Arizona, USA

**Keywords:** complete genome, plant pathogens, *Pseudomonas syringae* pv. *actinidiae*, China

## Abstract

Here, we report the complete genome sequence for *Pseudomonas syringae* pv. *actinidiae* strain Yunnan3.2, which was isolated from diseased kiwifruit grown in Yunnan province, China. The complete genome of Yunnan3.2 comprises a 6,564,315-bp chromosome with a GC content of 58.41% and a circular plasmid (74,466 bp).

## ANNOUNCEMENT

*Pseudomonas syringae* pv. *actinidiae* (*Psa*) causes bacterial canker of kiwifruit (*Actinidia deliciosa* and *A. chinensis*), one of the most devastating bacterial diseases of kiwifruit (1). Since *Psa* was first isolated in Japan (2), it has been found in several countries, including Korea, Italy, France, New Zealand, and Spain (1, 3–7). In China, this disease has been reported in multi-provinces including Sichuan, Hunan, Anhui, and Shaanxi (8–10). Here, we presented a high-quality, complete genome sequence of *Psa* strain Yunnan3.2.

*Psa* strain was isolated from diseased kiwifruit branches grown in Zhaotong (N27°, E103°), Yunnan province, China. Branch tissues were sterilized using 70% ethanol and ground with sterile water. The diluted solution was plated onto Luria-Bertani (LB) agar plate (11) and incubated at 28°C. Multiple single colonies were identified as *Psa* strain through PCR amplification using specific primer pair Psa-F/Psa-R (12). Among them, Yunnan3.2 strain was chosen for sequencing. A single colony of Yunnan3.2 strain was cultured in LB liquid medium for 12 h, and 5 mL of the bacterial suspension was centrifuged to collect bacterial cells. Then, the bacterial cells were submitted to Beijing Novogene Bioinformatics Technology Co., Ltd. (Beijing, China), for sequencing. Genomic DNA was extracted using STE method (13) and quantified with Qubit fluorometer (Thermo Scientific, USA). The whole genome of Yunnan3.2 was sequenced on both the PacBio platform and Illumina PE150 platform, using SMRT bell TM Template kit (version 2.0) and NEBNextUltra DNA Library Prep Kit for Illumina (NEB, USA), respectively. Low-quality reads were filtered using SMRT Link v8.0, and the filtered reads were assembled by software Canu (https://github.com/marbl/canu/, version 2.0) (14). A total of 762,396 reads with an N50 length of 7,919 bp and average read length of 4,972 bp were obtained through PacBio sequencing, which provided approximately 570-fold genome coverage. For Illumina sequencing, paired-end reads (2 × 150 bp), totaling 7,573,333 reads covering a total of 2.272 Gb clean data (Q20, 97.61; Q30, 93.43) with 424-fold genome coverage, were obtained. Pilon v1.23 was employed to correct the assembled genome using Illumina sequence data, resulting in a final genome with high accuracy (15). BLASTN was employed to assess the genomic loops (overlap >2 kb), and the overlapping parts were cut off (16). GeneMarkS was employed to predict and filter genes of the genome (17), while the publicly accessible version of the genome was annotated by NCBI Prokaryotic Genome Annotation Pipeline (PGAP) (18). All tools were run with default parameters unless otherwise specified.

**Fig 1:**
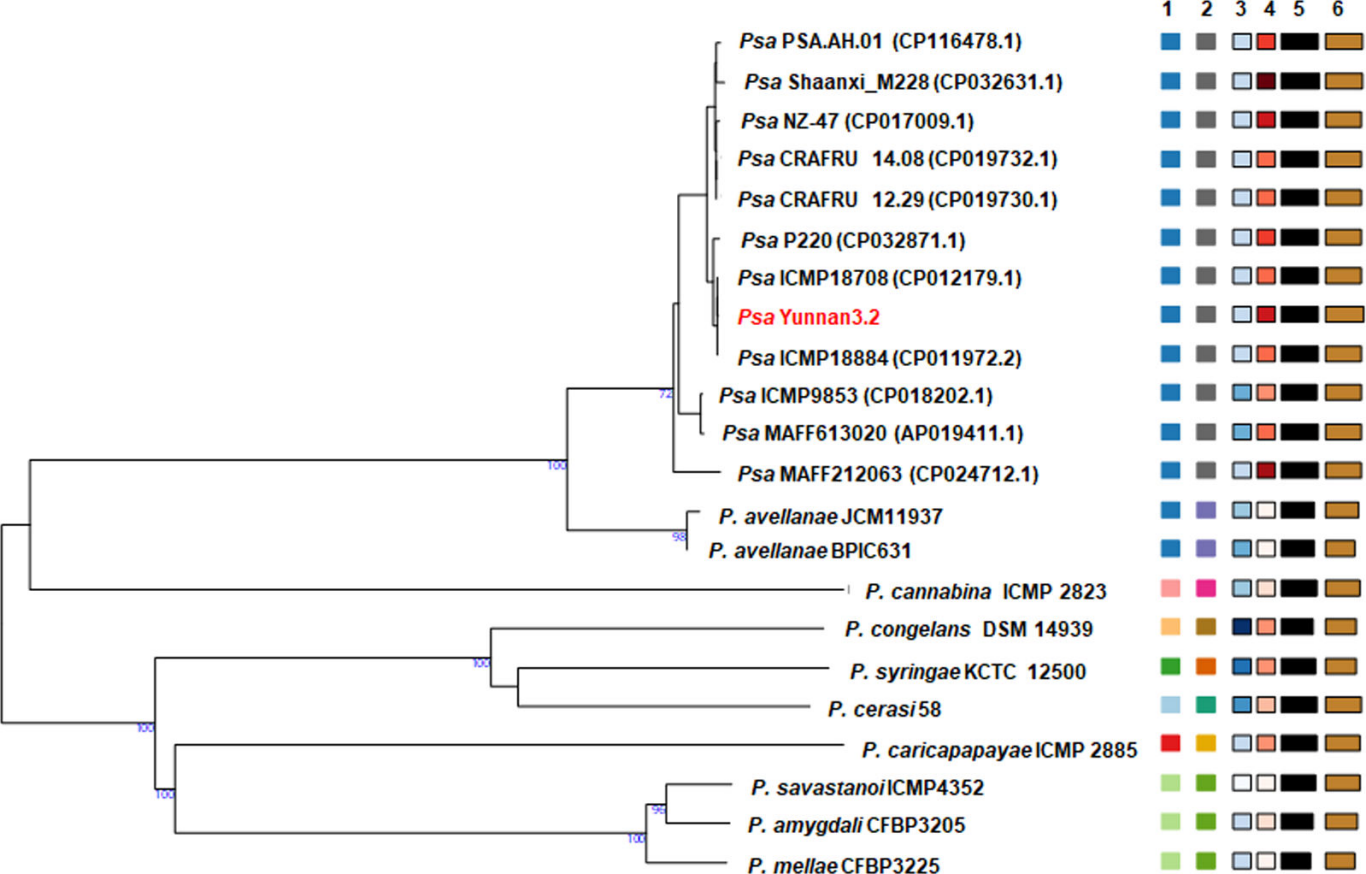
Phylogenetic tree of *Pseudomonas syringae* pv. *actinidiae* strain Yunnan3.2. The phylogenetic analysis was performed by the Type Strain Genome Server (TYGS) based on genome blast distance phylogeny (GBDP) distances calculated from genome sequences. The complete genome sequences of 10 *Pseudomonas* species were automatically deposited by TYGS. The genome sequences of the other 11 *Psa* strains were downloaded from the GenBank database. Notably, Yunnan3.2 strain is highlighted in red. The numbers below branches represent GBDP pseudo-bootstrap support values from 100 replications. Leaf labels are annotated according to (1) species cluster, (2) subspecies cluster, (3) percent G + C, (4) delta statistics, (5) genome size (in bp), and (6) protein count, respectively.

The genomic features for strain Yunnan3.2 are summarized in Table 1. The genome-based classification of strain Yunnan3.2 was conducted using the Type Strain Genome Server (TYGS) web server (19). Phylogenetic analysis revealed that Yunnan3.2 clustered together with two New Zealand *Psa* strains ICMP18708 (GenBank: CP012179) (20) and ICMP18884 (GenBank: CP011972) (21) (Fig. 1). The average nucleotide identity (ANI) values between strain Yunnan3.2 and ICMP18708, Yunnan3.2 and ICMP18884, and Yunnan3.2 and a Chinese *Psa* strain Shannxi_M228 (GenBank: CP048810) (22) were 99.95, 99.95, and 99.75, respectively. The analysis of ANI was performed by using EzBioCloud (https://www.ezbiocloud.net/tools/ani) (23).

**TABLE 1:** Genome annotation statistics of Yunnan3.2

Characteristic	Yunnan3.2^*a*^
Total bases (bp)	6,564,315
GC (%)	58.41
Genes	6,001
CDSs	5,918
Complete rRNAs (5S, 16S, 23S)	6, 5, 5
tRNA	63
ncRNA	4
Pseudo genes	480
CRISPR	44

^a^ Determined using PGAP.

## Data Availability

The assembled genome sequences described here have been deposited in GenBank under the accession numbers CP135283 (Yunnan3.2 chromosome) and CP135284 (plasmid pYUNNAN3.2). The raw genome sequence data obtained on the PacBio platform and Illumina PE150 platform have been deposited in SRA under the accession numbers SRR27335884 and SRR27332408, respectively.

## References

[1] Vanneste JL. 2017. The scientific, economic, and social impacts of the New Zealand outbreak of bacterial canker of kiwifruit (Pseudomonas syringae pv. actinidiae). Annu Rev Phytopathol 55:377–399. doi:10.1146/annurev-phyto-080516-035530 28613977

[2] Takikawa Y, Serizawa S, Ichikawa T, Tsuyumu S, Goto M. 1989. Pseudomonas syringae pv. actinidiae pv. nov.: the causal bacterium of canker of kiwifruit in Japan. Jpn J Phytopathol 55:437–444. doi:10.3186/jjphytopath.55.437

[3] Kim GH, Kim KH, Son KI, Choi ED, Lee YS, Jung JS, Koh YJ. 2016. Outbreak and spread of bacterial canker of kiwifruit caused by Pseudomonas syringae pv. actinidiae biovar 3 in Korea. Plant Pathol J 32:545–551. doi:10.5423/PPJ.OA.05.2016.0122 27904461 PMC5117863

[4] Renzi M, Copini P, Taddei AR, Rossetti A, Gallipoli L, Mazzaglia A, Balestra GM. 2012. Bacterial canker on kiwifruit in Italy: anatomical changes in the wood and in the primary infection sites. Phytopathology 102:827–840. doi:10.1094/PHYTO-02-12-0019-R 22713076

[5] Ferrante P, Scortichini M. 2009. Identification of Pseudomonas syringae pv. actinidiae as causal agent of bacterial canker of yellow kiwifruit (actinidia chinensis planchon) in central Italy. Journal of Phytopathology 157:768–770. doi:10.1111/j.1439-0434.2009.01550.x

[6] Vanneste JL, Poliakoff F, Audusseau C, Cornish DA, Paillard S, Rivoal C, Yu J. 2011. First report of Pseudomonas syringae pv. actinidiae, the causal agent of bacterial canker of kiwifruit in France. Plant Dis 95:1311. doi:10.1094/PDIS-03-11-0195 30731682

[7] Abelleira A, López MM, Peñalver J, Aguín O, Mansilla JP, Picoaga A, García MJ. 2011. First report of bacterial canker of kiwifruit caused by Pseudomonas syringae pv. actinidiae in Spain. Plant Dis 95:1583. doi:10.1094/PDIS-06-11-0537 30731983

[8] Wang Z, Tang X, Liu S. 1992. Identification of the pathogenic bacterium for bacterial canker on Actinidia in Sichuan. Journal of Southwest Agricultural University: unpaginated 14:500–503.

[9] He R, Liu P, Jia B, Xue S, Wang X, Hu J, Al Shoffe Y, Gallipoli L, Mazzaglia A, Balestra GM, Zhu L. 2019. Genetic diversity of Pseudomonas syringae pv. actinidiae strains from different geographic regions in China. Phytopathology 109:347–357. doi:10.1094/PHYTO-06-18-0188-R 30226424

[10] Liang YM, Zhang XY, Tian CM, Gao AQ, Wang PX. 2000. Pathogenic identification of Kiwifruit bacterial canker in Shaanxi. J Northwest For Univ 15:37–39.

[11] Hanahan D. 1983. Studies on transformation of Escherichia coli with plasmids. J Mol Biol 166:557–580. doi:10.1016/s0022-2836(83)80284-8 6345791

[12] Balestra GM, Taratufolo MC, Vinatzer BA, Mazzaglia A. 2013. A multiplex PCR assay for detection of Pseudomonas syringae pv. actinidiae and differentiation of populations with different geographic origin. Plant Dis 97:472–478. doi:10.1094/PDIS-06-12-0590-RE 30722225

[13] Lu SD. 1993. Modern molecular biology experimental techniques. 2nd ed, p 101–136. Peking Union Medical College Press, Beijing.

[14] Koren S, Walenz BP, Berlin K, Miller JR, Bergman NH, Phillippy AM. 2017. Canu: scalable and accurate long-read assembly via adaptive k-mer weighting and repeat separation. Genome Res 27:722–736. doi:10.1101/gr.215087.116 28298431 PMC5411767

[15] Walker BJ, Abeel T, Shea T, Priest M, Abouelliel A, Sakthikumar S, Cuomo CA, Zeng Q, Wortman J, Young SK, Earl AM. 2014. Pilon: an integrated tool for comprehensive microbial variant detection and genome assembly improvement. PLoS One 9:e112963. doi:10.1371/journal.pone.0112963 25409509 PMC4237348

[16] Chen Y, Ye W, Zhang Y, Xu Y. 2015. High speed BLASTN: an accelerated MegaBLAST search tool. Nucleic Acids Res 43:7762–7768. doi:10.1093/nar/gkv784 26250111 PMC4652774

[17] Besemer J, Lomsadze A, Borodovsky M. 2001. Genemarks: a self-training method for prediction of gene starts in microbial genomes. implications for finding sequence motifs in regulatory regions. Nucleic Acids Res 29:2607–2618. doi:10.1093/nar/29.12.2607 11410670 PMC55746

[18] Tatusova T, DiCuccio M, Badretdin A, Chetvernin V, Nawrocki EP, Zaslavsky L, Lomsadze A, Pruitt KD, Borodovsky M, Ostell J. 2016. NCBI prokaryotic genome annotation pipeline. Nucleic Acids Res 44:6614–6624. doi:10.1093/nar/gkw569 27342282 PMC5001611

[19] Meier-Kolthoff JP, Carbasse JS, Peinado-Olarte RL, Göker M. 2022. TYGS and LPSN: a database tandem for fast and reliable genome-based classification and nomenclature of prokaryotes. Nucleic Acids Res 50:D801–D807. doi:10.1093/nar/gkab902 34634793 PMC8728197

[20] Poulter RTM, Ho J, Handley T, Taiaroa G, Butler MI. 2018. Comparison between complete genomes of an isolate of Pseudomonas syringae pv. actinidiae from Japan and a New Zealand isolate of the pandemic lineage. Sci Rep 8:10915. doi:10.1038/s41598-018-29261-5 30026612 PMC6053426

[21] Templeton MD, Warren BA, Andersen MT, Rikkerink EHA, Fineran PC. 2015. Complete DNA sequence of Pseudomonas syringae pv. actinidiae, the causal agent of kiwifruit canker disease. Genome Announc 3:e01054-15. doi:10.1128/genomeA.01054-15 26383666 PMC4574371

[22] Ho J, Taiaroa G, Butler MI, Poulter RTM. 2019. The genome sequence of M228, a Chinese isolate of Pseudomonas syringae pv. actinidiae, illustrates insertion sequence element mobility. Microbiol Resour Announc 8:e01427-18. doi:10.1128/MRA.01427-18 30637393 PMC6318364

[23] Yoon SH, Ha SM, Lim JM, Kwon SJ, Chun J. 2017. A large-scale evaluation of algorithms to calculate average nucleotide identity. Antonie Van Leeuwenhoek 110:1281–1286. doi:10.1007/s10482-017-0844-4 28204908

